# Phenylpropanoid Glycoside and Phenolic Acid Profiles and Biological Activities of Biomass Extracts from Different Types of *Verbena officinalis* Microshoot Cultures and Soil-Grown Plant

**DOI:** 10.3390/antiox11020409

**Published:** 2022-02-17

**Authors:** Paweł Kubica, Adam Kokotkiewicz, Magdalena Anna Malinowska, Alicja Synowiec, Małgorzata Gniewosz, Shah Hussain, Muhammad Yaqoob, Günther K. Bonn, Thomas Jakschitz, Eman A. Mahmoud, Tarek K. Zin El-Abedin, Hosam O. Elansary, Maria Luczkiewicz, Halina Ekiert, Agnieszka Szopa

**Affiliations:** 1 Chair and Department of Pharmaceutical Botany, Faculty of Pharmacy, Medical College, Jagiellonian University, Medyczna 9, 30-688 Krakow, Poland; p.kubica@uj.edu.pl; 2 Chair and Department of Pharmacognosy, Faculty of Pharmacy, Medical University of Gdansk, Al. Gen. J. Hallera 107, 80-416 Gdansk, Poland; adamkokot@gumed.edu.pl (A.K.); mlucz@gumed.edu.pl (M.L.); 3 Organic Chemistry and Technology Department, Faculty of Chemical Engineering and Technology, Cracow University of Technology, Warszawska 24, 31-155 Cracow, Poland; magdalena.malinowska@pk.edu.pl; 4 Department of Food Biotechnology and Microbiology, Warsaw University of Life Sciences–SGGW, ul. Nowoursynowska 159c, 02-776 Warsaw, Poland; alicja_synowiec@sggw.edu.pl (A.S.); malgorzata_gniewosz@sggw.edu.pl (M.G.); 5 ADSI—Austrian Drug Screening Institute GmbH, Innrain 66a, A-6020 Innsbruck, Austria; shah.hussain@adsi.ac.at (S.H.); muhammad.yaqoob@adsi.ac.at (M.Y.); guenther.bonn@uibk.ac.at (G.K.B.); thomas.jakschitz@adsi.ac.at (T.J.); 6 Department of Food Industries, Faculty of Agriculture, Damietta University, Damietta 34511, Egypt; emanmail2005@yahoo.com; 7 Department of Agriculture & Biosystems Engineering, Faculty of Agriculture (El-Shatby), Alexandria University, Alexandria 21545, Egypt; drtkz60@gmail.com; 8 Plant Production Department, College of Food & Agriculture Sciences, King Saud University, Riyadh 11451, Saudi Arabia; helansary@ksu.edu.sa

**Keywords:** vervain, in vitro cultures, phenylpropanoid glycosides, phenolic acids, antioxidant activity, antibacterial properties

## Abstract

Different types of microshoot cultures (agar, stationary liquid, agitated, and bioreactors) of *Verbena officinalis* were optimized for biomass growth and the production of phenylpropanoid glycosides and phenolic acids. Using ultra-high performance liquid chromatography with high-resolution time-of-flight mass spectrometry, the presence of verbascoside, isoverbascoside, leucoseptoside A/isomers, and cistanoside D/isomer was confirmed in the methanolic extracts obtained from all types of in vitro cultures. The compound’s content was determined by ultra-high-performance liquid chromatography. The main metabolites in biomass extracts were verbascoside and isoverbascoside (maximum 4881.61 and 451.80 mg/100 g dry weight (DW)). In the soil-grown plant extract, verbascoside was also dominated (1728.97 mg/100 g DW). The content of phenolic acids in the analyzed extracts was below 24 mg/100 g DW. The highest radical scavenging activity was found in the biomass extract from agitated cultures, the most effective reducing power in agar culture extract, and the highest chelating activity in extract from bioreactor cultures. The extracts showed significantly stronger bacteriostatic and bactericidal activity against Gram-positive bacteria (minimum inhibitory concentration (MIC) of 0.3–2.2 mg/mL and minimum bactericidal concentration (MBC) of 0.6–9 mg/mL) than against Gram-negative bacteria (MIC 0.6–9 mg/mL, MBC of 0.6–18 mg/mL). The biomass extract from liquid stationary culture showed the strongest antibacterial activity, while the extract from soil-grown herb had the lowest.

## 1. Introduction

*Verbena officinalis* L. (Verbenaceae), also known as vervain and common verbena, is a cosmopolitan species found in Europe, Asia, Australia, North America, South America, and northern Africa [1,2,3,4]. Its herb (*Verbenae officinalis herba*) has long been used in traditional medicine for its anti-inflammatory, antimicrobial, secretolytic, and expectorant properties [5,6,7]. The latest studies on *V. officinalis* herb extracts have confirmed that this plant’s raw material is characterized by several valuable biological activities, including antioxidant, antibacterial, antifungal, anti-inflammatory, antiproliferative, analgesic, antidepressant, anticonvulsant, sedative, anxiolytic, gastroprotective, and insecticidal properties [5,8,9,10,11]. In 2008, a monograph entitled “Verbena herb” was included in the sixth edition of the European Pharmacopoeia [1]. In addition, *V. officinalis* herb has been reported by the European Food Safety Authority (EFSA) as food constituent which protect cells and tissues against oxidative damage [12] and included in the CosIng (Cosmetic Ingredient) database [2] as a raw material that can be used in official European phytotherapy as well as in the food and cosmetic industries. The valuable biological activity profiles of *V. officinalis* herbs can be mainly attributed to the presence of compounds from the group of iridoids, phenylpropanoid glycosides, polyphenols, and essential oils [5,6,13,14,15,16,17,18,19].

The major iridoids found in *V. officinalis* are verbenalin, hastatoside, and aucubin, while the dominant phenylpropanoid glycosides are verbascoside, isoverbascoside, and eukovoside. The main phenolic acids identified are chlorogenic acid, ferulic acid, protocatechuic acid, rosmarinic acid, and dicaffeoylquinic acid derivatives, and the flavonoids are kaempferol, apigenin, luteolin, scutellarein, and pedalitin. Essential oil is dominated by terpenoids. The herb contains also sterols, and carbohydrates [5,6,13,14,15,16,17,18,19].

The current European Pharmacopoeia (10th Edition) indicates that the verbenalin content in standardized *V. officinalis* raw material should be no less than 1.5% dry weight (DW) [3].

Our objects of interest are the in vitro cultures of *V. officinalis*. Previously, we demonstrated the biotechnological possibilities of producing some phenylpropanoid glycosides and phenolic acids in the biomass of undifferentiating *V. officinalis* callus and suspension cultures that were maintained in Erlenmeyer flasks, as well as in special balloon and stirred-tank bioreactors. In these studies, we investigated the influence of different plant growth regulators (PGRs), light conditions, and inoculum size on biomass growth and metabolic profile [19,20,21]. The results showed that verbascoside was produced at an extremely high level, with a maximum amount of 9.18 g/100 g DW found in the suspension cultured in stirred-tank bioreactors [20]. These results are plausible given the various biological properties of verbascoside, such as antioxidant, anti-inflammatory, cytoprotective, antibacterial, antiviral, and antiandrogen activities [22,23,24].

Encouraged by these promising results, we decided to initiate shoot-differentiated cultures, which could allow for higher production of secondary metabolites as observed in the shoot cultures of different plant species [25,26,27].

In this study, we tested the following four types of cultures: microshoot agar cultures, stationary liquid cultures in special Magenta™ vessels (Magenta LLC, Lockport, IL, USA), agitated cultures in Erlenmeyer flasks, and cultures in the commercially available temporary immersion systems—RITA^®^ bioreactors (Vitropic, Saint-Mathieu-de-Tréviers, France).

We aimed to provide a comprehensive insight into the qualitative and quantitative profiles of two metabolite groups—phenylpropanoid glycosides and phenolic acids—in in vitro-cultured biomass extracts and in comparison with the extracts of soil-grown plants, using high-performance liquid chromatography with diode-array detection (HPLC-DAD) and ultra-high performance liquid chromatography with ultra-high resolution time-of-flight mass spectrometry (UHPLC-HR-QTOF-MS). In addition, the biological activities of the studied extracts were evaluated to determine their antioxidant capacity using three different tests (DPPH (1,1-diphenyl-2-picryl-hydrazyl) assay, reducing power assay, and ferrous ion (Fe^2+^) chelating activity assay). Furthermore, we investigated the antibacterial effects of the extracts on Gram-positive and Gram-negative bacterial strains. All the results were obtained in comparison with soil-grown plant extract.

## 2. Materials and Methods

### 2.1. Plant Material

The aerial parts of *V. officinalis* L. (Verbenaceae) at the flowering stage were acquired from Garden of Medicinal Plants, Faculty of Pharmacy, Jagiellonian University, Medical College, Kraków (Poland). The plant material was harvested in 2017 and air-dried at room temperature.

### 2.2. Initiation of In Vitro Cultures

In vitro microshoot cultures of *V. officinalis* L. were established from the shoot fragments of plants growing in the Garden of Medicinal Plants, Faculty of Pharmacy, Jagiellonian University, Medical College, Kraków (Poland). The plant fragments were placed on Murashige and Skoog (1962, MS) agar medium supplemented with 1 mg/L 6-benzylaminopurine and 1 mg/L indole-3-butyric acid. Microshoot cultures were obtained by subculturing the explants at 4-week intervals, but due to high callus growth and low numbers of new microshoots with signs of chlorosis, the cultures were transferred to Schenk and Hildebrandt (SH) medium (1972) [28] solidified using agar, which was supplemented with 500 mg/L of inositol and two cytokinins, namely, 6-γ,γ-dimethylallylaminopurine (2iP, 2 mg/L) and thidiazuron (TDZ, 0.22 mg/L). The medium was selected based on the results of previous experiments on microshoot cultures of other plant species. During growth cycles, this medium was found to be effective not only for the cultivation of microshoots in a continuous system but also for the multiplication of microshoots which could be a good source of biological material for further experiments. Cultures were maintained under artificial light conditions (88 ± 8 mol × m^−2^ × s^−1^; Philips-Flora TL-D 35W/33 fluorescent lamps, Philips, France), at a photoperiod of 17 h/24 h. The temperature was maintained at 23 ± 1 °C. Culture was carried out for 21 days.

### 2.3. Experimental In Vitro Cultures

All experimental biomasses, agar (Vo-A), stationary liquid (Vo-LS), agitated (Vo-LA), and bioreactor cultures (Vo-RITA) were maintained on SH media supplemented with 500 mg/L inositol, 2 mg/L 2iP, and 0.22 mg/L TDZ. The light intensity during the 17h/24h photoperiod was set at 88 ± 8 mol × m^−2^ × s^−1^. The cultures were maintained at a temperature of 23 ± 1 °C.

Agar cultures were initiated using 1 g of microshoots on 30 mL of solidified medium and maintained in 28-day growth cycles. Stationary liquid cultures were maintained in Magenta vessels using 1 g of microshoots in 30 mL of liquid medium for 28 days. Agitated cultures were maintained in Erlenmeyer flasks using 1 g of microshoots in 30 mL of liquid medium for 28 days on an orbital shaker at 120 rpm (Innova platform shaker, New Brunswick Scientific, Enfield, CT, USA). Bioreactor cultures were maintained in RITA bioreactors (a temporary immersion system) during 21-day growth cycles. The initial biomass was placed in individual bioreactors in the same quantitative ratio (biomass/medium), as in agar, stationary liquid, and agitated cultures. The amount of liquid medium added in each bioreactor was 200 mL. The working cycle to bioreactors consisted of 5 min of aeration and 85 min of resting time, with 16 cycles per day.

The biomasses harvested from all types of cultures were washed with water, and then both biomass and media samples were lyophilized (GT 2 apparatus, Finn-Aqua Santasalo-Sohlberg, Tuusula, Finland).

### 2.4. Extraction

#### 2.4.1. For HPLC–DAD Analyses

For the analysis of phenylpropanoid glycosides, the methanolic extracts were prepared using 300 mg of plant material (lyophilized biomass of each in vitro culture and dried herb of a soil-grown plants). Extraction was carried out in ultrasonic bath (Polsonic 3, Warsaw, Poland) with methanol. Each sample was extracted five times with 3-mL portions of solvent (30 min per extraction). After extraction, five extracts of each sample were pooled together and centrifuged. The resulting supernatant was filtered using a syringe filter (0.22 µm; Millex-GP Syringe Filter Unit, Millipore, Burlington, MA, USA).

Analysis of phenolic acids was carried out in samples prepared from 10-mL portions of each extract dried in crystallizers to a constant weight and dissolved in 2 mL of methanol. The extracts were filtered through a syringe filter (0.22 µm, Millex-GP Filter Syringe Unit, Millipore).

In addition, culture media (20 mL) collected after each experiment were lyophilized, dissolved in 1 mL of methanol, and filtered using syringe filters (0.22 µm, Millex-GP Filter Syringe Unit, Millipore).

#### 2.4.2. For UHPLC-HR-QTOF-MS Qualitative Analyses

For qualitative analyses, two extracts were prepared: from in vitro cultures (represented by agar microshoot cultures) and from the herb of soil-grown plants. Briefly, 500 mg of material was mixed with 5 mL of methanol. The resulting mixture was sonicated for 30 min at room temperature. The extraction mixture was centrifuged at 7000 rpm for 10 min, and the supernatant was filtered through a 0.2-µm polytetrafluoroethylene syringe filter. The filtered solution was diluted 200-fold with methanol prior to instrumental analysis.

#### 2.4.3. For Biological Investigations

For biological analyses, the samples (lyophilized biomass of each in vitro culture and dried herb of a soil-grown plant).were extracted twice in methanol in an ultrasonic bath (Polsonic 3, Warsaw, Poland) at a sample/methanol ratio of 1/40. The extracts were centrifuged, and the resulting supernatant was placed in crystallizers and dried at room temperature to a constant mass.

### 2.5. Chromatographic Analyses—HPLC–DAD

#### 2.5.1. Phenylpropanoid Glycosides

Analyses of verbascoside and isoverbascoside (phenylpropanoid glycosides) were carried out using the HPLC–DAD method as described by Schönbichler et al. [29]. The column used for the analyses was Kinetex C18 analytical column (150 × 4.6 mm, 2.7 µm; Phenomenex, Torrance, CA, USA). The mobile phase used for gradient program consisted of the following two solvents: 0.1% trifluoroacetic acid and acetonitrile. The analyses of phenylpropanoid glycosides were carried out at a wavelength of 330 nm. Qualitative and quantitative calculations were performed by comparing the UV-DAD spectra and retention times (Rt) of compounds with those of commercially available standards (ChromaDex, Los Angeles, CA, USA). Quantification was carried out based on calibration curves.

#### 2.5.2. Phenolic Acids

Analyses of phenolic acids were carried out using the HPLC–DAD method as described by Ellnain-Wojtaszek and Zgórka [30,31]. The column used for the analyses was Purospher RP-18e analytical column (4 × 250 mm, 5 mL; Merck, Darmstadt, Germany). The mobile phase used for gradient program consisted of the following two solvents: methanol with 0.5% acetic acid (1:4, *v*/*v*) and methanol. Calculations were performed at a wavelength of 254 nm. The compounds in the investigated extracts were identified by comparing their UV-DAD spectra and Rt values with those of commercially available standards. Quantification was carried out based on calibration curves.

#### 2.5.3. Chromatographic Analyses—UHPLC-HR-QTOF-MS

Analyses were carried out on Thermo Scientific Dionex UltiMate 3000 coupled with a Maxis Impact Ultra-High Resolution TOF-MS system (Bruker^®^ Daltonics, Bremen, Germany). The analytical column (heated to 45 °C during analysis) used was Agilent RRHD Zorbax C18 (2.1 × 100 mm, 1.8 μm) The mobile phase consisted of the following two solvents: acetonitrile (B) and 0.01% formic acid in water (A). The flow rate was set at 0.4 mL/min. The LC gradient program was as follows: Min/B%: 0/5, 1/25, 10/40, 12/100, 14/100, 14.5/5, and 16/5. The temperature of the sampler was maintained at 4 °C. The injection volume was 2 μL.

The parameters set for electrospray ionization in positive mode were as follows: nitrogen nebulizer gas pressure: 3 bar; drying gas flow rate: 12 L/min; end plate offset: 500 V; capillary voltage: +4500 V; dry temperature: 200 °C; funnel 1 RF: 200 Vpp and funnel 2 RF: 150 Vpp; CID energy: 0 eV; hexaploe RF: 50 Vpp; quadrupole ion energy: 5 eV; and low-mass filtering: 50 m/z. The collision cell parameters were as follows: collision energy: 10 eV; collision RF: 500 Vpp; transfer time: 50 µS; and prepulse storage time: 6 µs.

Data analysis was performed using DataAnalysis 4.2 software (Bruker Daltonics, Bremen, Germany).

#### 2.5.4. Determination of Total Phenolic Content

The total phenolic content of the extracts was determined using the Folin–Ciocalteu method. The calibration curve of gallic acid was used as a reference for the analysis [32]. The sample for the analysis was prepared by mixing 100 mL of the sample solution with 0.2 mL of Folin–Ciocalteu reagent, 2 mL of H_2_O, and 1 mL of 15% Na_2_CO_3_. After 2 h of incubation at room temperature, the absorbance of the samples was measured at a wavelength of 765 nm using a Nanocolor UV/VIS spectrophotometer (Macherey-Nagel, Düren, Germany). The total phenolic content was estimated as gallic acid equivalent (GAE) and expressed in mg GAE/g extract ± standard deviation (SD). The calibration curve was based on six different concentrations of gallic acid: 0.0625 mg/mL, 0.125 mg/mL, 0.25 mg/mL, 0.5 mg/mL, 1.0 mg/mL, and 2 mg/mL. Data were obtained from the average of three determinations.

### 2.6. Antioxidant Activity

#### 2.6.1. Free Radical Scavenging Activity

The free radical scavenging activity of the extracts was determined using the DPPH (1,1-diphenyl-2-picryl-hydrazyl) method [32]. Briefly, an aliquot (0.5 mL) of each extract at different concentrations (0.0625–2 mg/mL) was added to 3 mL of freshly prepared methanol–DPPH solution (0.1 mM). After 20 min of initial mixing, the absorbance of the solution was measured at a wavelength of 517 nm, using a Nanocolor UV/VIS spectrophotometer (Macherey-Nagel). Butylated hydroxytoluene (BHT) at the same concentrations as the extract was used for reference. The results were averaged from three independent experiments and reported as mean radical scavenging activity (%) ± SD and mean 50% inhibitory concentration (IC_50_) ± SD.

#### 2.6.2. Reducing Power Assay

The reducing power of *V. officinalis* extracts was tested by the spectrophotometric measurement of Fe^3+^-to-Fe^2+^ transformation [32] using a Nanocolor UV/VIS spectrophotometer (Macherey-Nagel). Briefly, different concentration of each sample (0.0625–2 mg/mL) in 1 mL of methanol were mixed with 2.5 mL of 0.2 M phosphate buffer (pH 6.6) and 2.5 mL of 1% potassium ferricyanide [K3Fe(CN)6]. After incubation at 50 °C for 20 min, the solution was cooled rapidly in ice, mixed with 2.5 mL of 10% trichloroacetic acid, and centrifuged at RCF 1100 for 10 min. 2.5 mL of the resulting supernatants were mixed with 2.5 mL of deionized water and 0.5 mL of 0.1% freshly prepared ferric chloride (FeCl3) and left at room temperature for 10 min. The absorbance values of the samples were determined at 700 nm. Ascorbic acid and butylated hydroxytoluene (BHT), at the same concentrations as the tested samples, were used as the reference. The experiments were run in triplicates. The results were expressed as the mean absorbance values ± SD and ascorbic acid equivalents (ASE/mL) ± SD. The calibration curve equation obtained for ascorbic acid was y = 0.876x + 0.5833 (R^2^ = 0.9788).

#### 2.6.3. Ferrous Ions (Fe^2+^) Chelating Activity

The Fe^2+^ chelating activity of the extracts tested was measured by the evaluation of Fe^2+^–ferrozine complex establishment according to the previously described method [32]. 1 mL of each extract at different concentrations of each sample (0.0625–2 mg/mL) were mixed with 0.5 mL of methanol and 0.05 mL of 2 mM FeCl_2_. The addition of 0.1 mL of 5 mM ferrozine initiated the complex formation. The obtained mixtures were shaken vigorously and left aside for 10 min at room temperature. Then, the absorbance values of the samples were measured at 562 nm using a Nanocolor UV/VIS spectrophotometer (Macherey-Nagel). The solution of ethylenediaminetetraacetic acid (EDTA) was used as a reference. The experiments were run in triplicates and the and the results obtained were presented as the average values and reported as mean inhibition of the Fe^2+^–ferrozine complex formation (%) ± SD and IC_50_ ± SD.

### 2.7. Antibacterial Activity

#### 2.7.1. Bacterial Strains and Preparation of Inoculum

The antibacterial activity of all extracts was tested against four strains of Gram-positive bacteria (*Staphylococcus epidermidis* ATCC 12228, *Staphylococcus aureus* ATCC 25923, *Bacillus cereus* ATCC 11778, and *Listeria monocytogenes* NIPH-NIH 17/11) and eight strains of Gram-negative bacteria (*Yersinia enterocolitica* O3 NIPH-NIH 383/11, *Pseudomonas aeruginosa* ATCC 27853, *Klebsiella pneumoniae* ATCC 13883, *Proteus mirabilis* ATCC 35659, *Shigella sonnei* NIPH-NIH, *Salmonella enterica* subsp. *enterica* serovar Enteritidis ATCC 13076, *Enterobacter aerogenes* ATCC 13048, and *Escherichia coli* ATCC 25922).

The strains were obtained from the American Type Culture Collection (ATCC, Manassas, VA, USA), and clinical isolates from the National Institute of Public Health—National Institute of Hygiene (NIPH—NIH, Warsaw, Poland).

The bacterial strains were cultured on nutrient agar for 24 h at 37 °C. The inocula were diluted to approximately 1 × 10^8^ cfu/mL using 0.85% NaCl (*w*/*v*).

#### 2.7.2. MIC and MBC Determination

The MIC (minimum inhibitory concentration) and MBC (minimum bactericidal concentration) of the extracts were determined by serial microdilution [33,34]. For this purpose, two series of dilutions (ranging from 18.0 to 0.15 mg/mL) were prepared. The extracts were diluted with Mueller–Hinton Broth medium. Bacterial inocula containing 5 × 10^5^ cfu/mL of cells were added to 96-well plates (250 µL to each well). A well without extract served as the positive control, while the negative control consisted of medium with the tested bacterial strain and no extract. The plates were incubated at 37 °C for 20 h. Then, 25 µL of sterile 0.02% (*m/v*) resazurin solution was added to each well and the plates were incubated again at 37 °C for 2 h. Resazurin acts as an indicator of bacterial growth. The color change (from purple to pink) in wells was assessed by comparing with the control [35].

The MIC value (expressed as mg/mL) was defined as the lowest concentration of extract in which no bacterial growth was observed. This analysis was repeated three times.

For the determination of MBC, 100 µL of mixture from each well in which no bacterial growth was reinoculated onto Mueller–Hinton agar medium. After incubation at 37 °C for 24 h, the plates were examined for the growth of colonies. The MBC value (expressed in mg/mL) was defined as the lowest concentration of the extract that resulted in complete inhibition of bacterial growth.

The percentage value of the antibacterial activity of the extracts was determined based on MIC values (A) [36], which is calculated using the following formula: A% = (100 × number of strains inhibited by the examined extract)/(total number of tested strains).

For the results expressed as the mean value of three independent measurements, standard deviation was also calculated for each experiment.

## 3. Results and Discussion

### 3.1. Influence of Cultivation Mode on Microshoot Appearance and Biomass Increments

To evaluate the efficacy of the used experimental models for maintaining in vitro cultures, the growth of the microshoot biomass was determined as a growth index (Gi), which was calculated based on the initial and final mass of microshoots. The Gi parameter is a measure of the increase in tissue mass (%) after the growth period.

$$\mathrm{Gi}\;\left(\%\right)=\frac{\mathrm{final}\;\mathrm{mass}\;\left[g\times l^{-1}\right]-\mathrm{initial}\;\mathrm{mass}\left[g\times l^{-1}\right]\;}{\mathrm{initial}\;\mathrm{mass}\left[g\times l^{-1}\right]}\times 100$$

**Figure 1:**
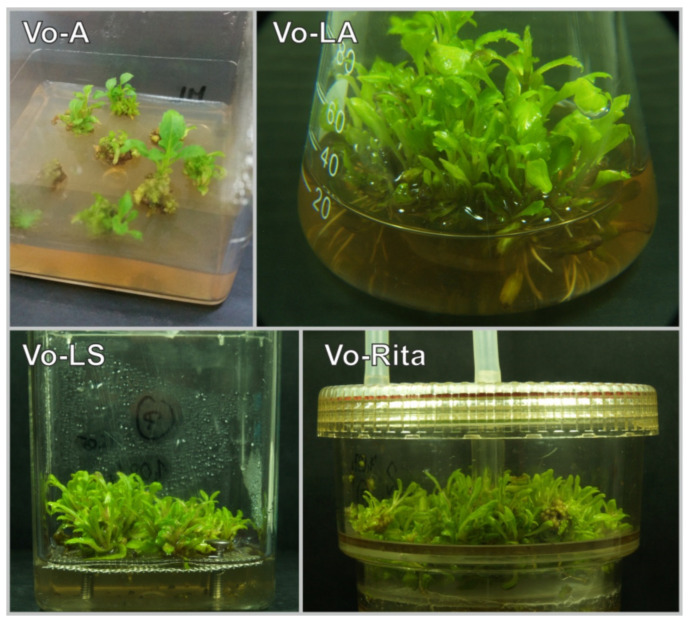
Macroscopic appearance of experimental biomass in the investigated types of *V. officinalis* cultures; agar (Vo-A), stationary liquid (Vo-LS), agitated (Vo-LA), and bioreactor cultures (Vo-RITA).

**Figure 2:**
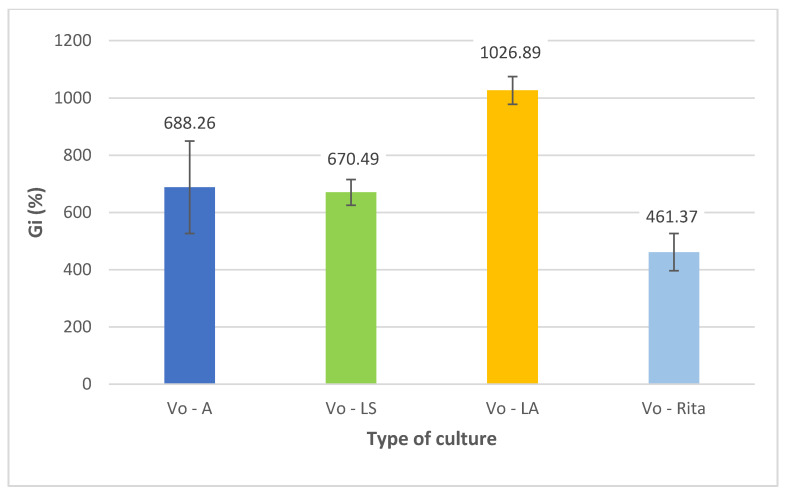
Comparison of the Gi values of *V. officinalis* microshoots grown in the investigated types of *V. officinalis* cultures; agar (Vo-A), stationary liquid (Vo-LS), agitated (Vo-LA), and bioreactor cultures (Vo-RITA).

In the Vo-A cultures, the biomass had an intense green color, the microshoots were gradually elongated, and new leaves were formed during the growth period (Figure 1). Microshoots that were immersed in the medium did not show necrosis due to hypothetical hypoxia and even exhibited dynamic growth. Primordia with direct access to air reproduced fully developed leaves. Spontaneous rhizogenesis was also observed; however, the underlying mechanism could not be determined at this stage of the experiment. The Vo-A cultures showed a higher increase in growth. The Gi value reached 688.26%, which is large for highly organized tissues such as microshoots [37] (Figure 2). Furthermore, the amount of tissue mass obtained from Vo-A cultures was significant (fresh weight (FW) = 281.4 g/L; DW = 20.3 g/L).

The results obtained for the investigated medium and PGRs were satisfactory, and therefore no study was conducted on other variants of the medium to improve the growth parameters of Vo-A cultures.

Transfer of *V. officinalis* microshoot cultures from the agar medium to liquid agitated conditions resulted in a significant improvement in growth parameters (Gi = 1027%, FW = 393.2 g/L, DW = 22.6 g/L) (Figure 2). A larger amount of biomass was obtained, and the medium used was also found to be more effective (Gi was almost 1.5-fold higher than in the Vo-A culture). Similar to Vo-A culture, Vo-LA culture was characterized by an intense green color with no necrotic elements (Figure 1). Moreover, further development of primordial structures into fully developed leaves was observed, with a typical morphology of a soil-grown plant.

The change of the type of culture to stationary liquid (Magenta vessel) culture or bioreactor culture (RITA bioreactors) (Figure 1) led to the deterioration of growth parameters. In both cases, the Gi values of the cultivated biomass were lowered by about half (Figure 2).

In our previous study, the undifferentiated cultures of *V. officinalis* (callus tissue) reached a greater Gi. In these cultures, the highest Gi value (1561%) was determined after 2 weeks of the growth period [20].

Some Gi values reported by other authors based on studies with differentiated cultures were higher or comparable with those of our current study. The Gi value of 1959% was observed for *Schisandra chinensis* microshoot agitated cultures, but for 60 days of the growth period [38]. However, under other conditions, for microshoots maintained in RITA bioreactors over 60 days, the Gi value of *S.* *chinensis* reached lower Gi 1078% [39]. In *Nasturtium officinale* agitated cultures maintained for 20 days, the maximal Gi value was 1048% [40]. For *Rhododendron tomentosum* shoot cultures maintained in bioreactors, the Gi values were several times smaller—in a RITA bioreactor, the Gi value reached 280% and in a spray-glass bioreactor, the Gi value reached 250% [41].

### 3.2. Target Metabolic Profiles

In order to compare the qualitative composition of in vitro microshoots and soil-grown plants, the UHPLC-HR-QTOF-MS assay was performed. The main compounds identified in the agar microshoot culture extract were verbascoside, isoverbascoside, leucoseptoside A/isomers, and cistanoside D/isomer (Table 1). Significant differences in the content of these compounds were observed between the analyzed *V. officinalis* herb extract and the microshoot culture extract. The relative abundance of verbascoside and isoverbascoside was higher in in vitro culture extracts. Similarly, leucoseptoside A/isomers and cistanoside D/isomer were also relatively higher in in vitro cultures.

Using the available standards, we performed a quantitative analysis of phenylpropanoid glycosides (verbascoside and isoverbascoside) and phenolic acids using the HPLC–DAD method on all investigated extracts from in vitro and soil-grown plants.

The results showed that the amounts of phenylpropanoid glycosides varied depending on the type of culture. The concentration of verbascoside was high in the biomass extracts of all tested types of cultures and ranged from 4722.20 to 4881.61 mg/100 g DW. On the other hand, the amounts of isoverbascoside were lower and ranged from 264.90 to 451.80 mg/100 g DW. The maximal total content of both phenylpropanoid glycosides (5333.42 mg/100 g DW) was observed in the biomass extracts from Vo-LA (Table 2).

In the extracts from soil-grown plant material (Vo-in vivo), the average content of verbascoside during blooming period was determined at 1728.97 mg/100 g DW and that of isoverbascoside at 78.34 mg/100 g DW (Table 2). In all the investigated extracts from in vitro cultures, the amounts of verbascoside and isoverbascoside were higher compared to the extracts from plant material. The content of verbascoside was 2.79-fold higher in Vo-A extract, 2.76-fold higher in Vo-LS extract, 2.82-fold higher in Vo-LA extract, and 2.73-fold higher in Vo-RITA extract. The content of verbascoside decreased in the following order: Vo-LA > Vo-A > Vo-LS > Vo-RITA >> Vo-in vivo. The amounts of isoverbascoside were also higher in extracts from in vitro cultures (4.22-fold in Vo-A extract, 3.38-fold in Vo-LS extract, 5.77-fold in Vo-LA extract, and 3.40-fold in Vo-RITA extract) than in plant extract. The content of isoverbascoside decreased as follows: Vo-LA > Vo-A > Vo-RITA ≈ Vo-LS >> Vo-in vivo.

In all the extracts obtained from in vitro cultures, only two phenolic acids, ferulic acid and protocatechuic acid, were detected. The level of ferulic acid did not exceed 23.7 mg/100 g DW and that of protocatechuic acid did not exceed 8.4 mg/100 g DW. The highest concentration of these two compounds was detected in the extracts from Vo-LA cultures (Table 3).

Compared to in vitro culture extracts, the herb extracts were richer in phenolic acids in terms of both quality and quantity. The plant extracts contained three phenolic acids, namely, ferulic, protocatechuic, and rosmarinic acids (29.8, 25.8, and 2.5 mg/100 g DW, respectively) (Table 3). The total content of phenolic acids decreased in the following order: Vo-in vivo > Vo-LA > Vo-LS >> Vo-RITA > Vo-A.

As shown in Table 3, the total phenolic content varied from 122.93 mg GAE/g extract (Vo-LA) to 163.58 mg GAE/g extract (Vo-A) and decreased in the following order: Vo-A > Vo-LS > Vo-in vivo > Vo-RITA > Vo-LA. The extracts from lyophilized culture media have only traces amounts of metabolites.

The chemical profiles of the *V. officinalis* microshoot cultures significantly differed from those of the callus cultures determined in our earlier study [20]. The obtained results show that microshoot cultures accumulate a smaller amount of verbascoside than all callus cultures grown in different systems (agar, agitated, and bioreactor cultures) [20]. The microshoot cultures produced up to a 1.88-fold (max. 4881.61 mg/100 g DW in Vo-LA) lower amount of verbascoside than the in vitro callus tissue cultures [20]. The highest amount of isoverbascoside estimated in microshoot cultures (451.80 mg/100 g DW in Vo-LA) was 1.35-fold lower than that confirmed in *V. officinalis* callus agar cultures (max. 609.26 mg/100 g DW), but was higher compared to the extracts from suspension cultures maintained in balloon bioreactors (306.40 mg/100 g DW) and stirred-tank bioreactors (339.91 mg/100 g DW) [20].

The metabolic pathways involved in the formation of phenylpropanoid glycosides, which were found to be dominant in the investigated in vitro microshoot cultures, were less active than those in callus cultures. The production of phenylpropanoid glycosides in microshoot cultures was enhanced compared to the soil-grown plant. A similar phenomenon was confirmed by other authors in the cultures of *Rehmannia elata* [42], *Harpagophytum procumbens* [43], *Castilleja tenuiflora* [44], and *Plantago lanceolata* [45]. In other studies, the content of verbascoside determined in different types of in vitro cultures was influenced by biotechnological approaches such as the addition of precursors or elicitors, genetic transformations, and maintenance of cultures in bioreactors [22]. The highest concentrations of phenylpropanoid glycosides (mostly verbascoside) were detected in in vitro microshoot cultures of *Syringa vulgaris* (16 g/100 g DW) [46]. Very high concentrations of verbascoside were reported in suspension cultures of *Buddleja cordata* (11.6 g/100 g DW) [47], *Plantago media* (9.16 g/100 g DW) [48], and cultures of *Cistanche salsa* (689 mg/L) [49] and *H. procumbens* maintained in pulse-aerated column bioreactors (165.42 mg/L/day) [50]. Depending on the cultivation method, high levels of verbascoside (from 2.2 to 6.0 g/100 g DW) as well as high levels of isoverbascoside (maximum content: from 0.8 to 1.8 g/100 g DW) were obtained in the hairy root cultures of *Rehmannia glutinosa* [51,52,53]. In the microshoot cultures of *Scutellaria baicalensis* maintained on MS medium, the content of verbascoside reached 830.9 mg/100 g DW [54], in the microshoot cultures of *Scutellaria altissima* it was 0.5 g/100 g DW [55] and in microshoot cultures of *Scutellaria lateriflora* maximal content on MS medium was 381.73 mg/100 g DW [56]. In the cell suspension culture of *Scutellaria alpina*, the content of verbascoside was 2.7 g/100 g DW [57].

Our results indicate that tissue differentiation in *V. officinalis* does not promote the accumulation of phenylpropanoid glycosides. The comparative analysis of the qualitative composition of extracts from microshoot cultures with the previously studied extracts from the in vitro cultures of callus tissue [20] revealed that in vitro microshoot cultures produced a lower variety of phenolic acids. The shoot cultures lacked chlorogenic, vanillic, caffeic, and rosmarinic acids [20]. In the soil-grown plant extracts, rosmarinic acid was present in a small amount (2.53 mg/100 g DW), while in the extracts from callus tissue maintained in vitro, it was found to be the dominant phenolic acid (up to 26.34 mg/100 g DW). On the other hand, in the microshoot cultures, this compound was completely absent, which clearly shows the metabolic differences between various types of in vitro cultures and soil-grown plants.

### 3.3. Biological Activities of the Studied In Vitro Cultures and Soil-Grown Plants

#### 3.3.1. Antioxidant Activity

The primary antioxidant activity of the tested extracts was examined using the DPPH test and reducing power assay, while the secondary antioxidant properties were determined by measuring the chelating activity of extracts [20].

**Figure 3:**
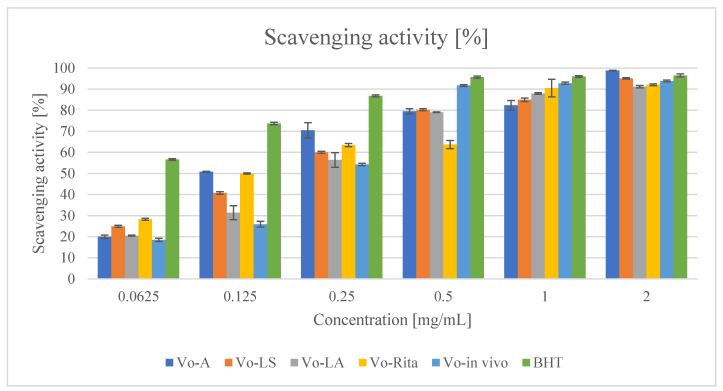
Free radical scavenging activity of extracts of *V. officinalis* in vitro cultures; agar (Vo-A), stationary liquid (Vo-LS), agitated (Vo-LA) and bioreactor cultures (Vo-RITA) and of plant material (Vo-in vivo). Values are expressed as mean ± SD (n = 3).

The results of the DPPH assay showed that all the tested extracts exhibited significant radical scavenging activity. The activity of 2 mg/mL concentrated extracts ranged from 91.16% (Vo-LA) to 98.82% (Vo-A) (Figure 3). Among the extracts, Vo-A extract was found to be the most effective, as was also confirmed by its IC_50_ value (0.081 mg/mL), and most importantly, the extract exhibited high efficiency at lower concentrations (91.97% at a dose of 0.50 mg/mL), which was comparable to the activity of BHT at the same concentration (95.71%). A comparison of IC_50_ values showed that the scavenging activity of extracts and standard decreased in the following order: BHT > Vo-A > Vo-RITA > Vo-LS > Vo-in vivo > Vo-LA (Table 4).

**Figure 4:**
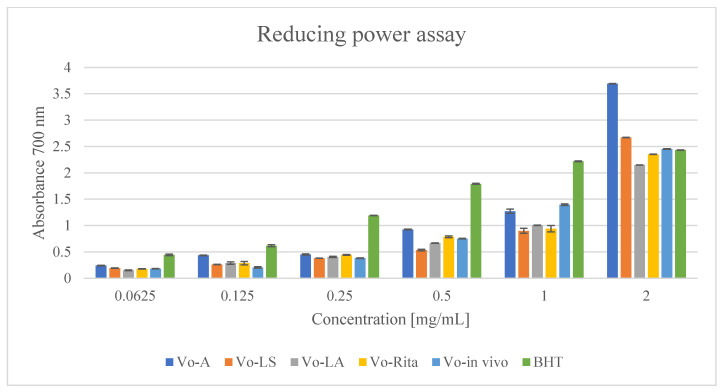
Reducing power of extracts of *V. officinalis* in vitro cultures; agar (Vo-A), stationary liquid (Vo-LS), agitated (Vo-LA) and bioreactor cultures (Vo-RITA) and of plant material (Vo-in vivo). Values are expressed as mean ± SD (n = 3).

The results of the reducing power assay showed that the extracts had good reducing power. The reducing power of extracts increased with an increase in their concentrations. The extracts from Vo-A, Vo-LS, and Vo-in vivo cultures exhibited the strongest activity. At a concentration of 2 mg/mL, the extracts showed a reducing power (ASE/mL) of 4.215, 3.048, and 2.800, respectively (Figure 4 and Table 4), which was found to be higher than that of BHT (2.776 ASE/mL). A comparison of the ASE/mL values showed that the reducing power of extracts and standards decreased in the following order: Vo-A > Vo-LS > Vo-in vivo > BHT > Vo-RITA > Vo-LA (Figure 4 and Table 4).

**Figure 5:**
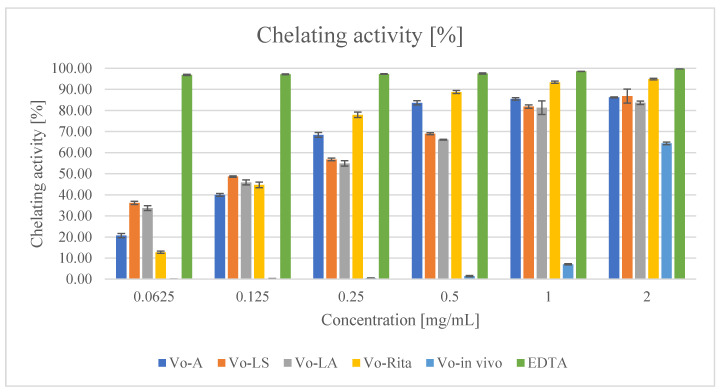
Ferrous ion (Fe^2+^) chelating activity of extracts of *V. officinalis* in vitro cultures; agar (Vo-A), stationary liquid (Vo-LS), agitated (Vo-LA) and bioreactor cultures (Vo-RITA) and of plant material (Vo-in vivo). Values are expressed as mean ± SD (n = 3).

The results of the Fe^2+^ chelating activity assay showed that all the tested extracts from in vitro cultures interfered with the formation of the ferrous–ferrozine complex starting from a concentration of 0.0625 mg/mL (33.72% for Vo-LA to 36.18% for Vo-LS), while EDTA showed a very high inhibitory activity (96.87%). Among the tested extracts, Vo-in vivo extract showed the lowest chelating ability (0.53% at 0.25 mg/mL) (Table 4 and Figure 5). At the maximum tested dose of 2 mg/mL, Vo-RITA showed the most efficient chelating activity, but it was slightly lower than that of the standard EDTA (94.86% and 99.80%, respectively). According to the calculated IC_50_ values, Vo-RITA was the most effective extract (IC_50_ = 0.034 mg/mL), and the metal chelating effect of the extracts and EDTA decreased in the following order: EDTA > Vo-RITA > Vo-LS > Vo-A > Vo-LA > Vo-in vivo (Table 4 and Figure 5).

The evaluation of *V. officinalis* antioxidant activity shows the significant potential of this raw material as an efficient protection against free radicals and reactive oxygen species. The results obtained are consistent with our previous studies of *V. officinalis* biomass extract activity evaluation [19]. The total phenolic content in previous experiments varied from 126.55 mg GAE/g extract to 189.91 mg GAE/g extract, which is in line with the presented results (122.93–163.58 mg GAE/g extract). The polyphenolic concentrations shown for *V. officinalis* are relatively high contents of these compounds in relation to other natural plant sources [58,59,60]. The scavenging ability results also correspond to the previous published (IC_50_ values from 0.110 to 0.137 mg/mL [19] and 0.069 mg/mL for the iridoids fraction of *V. officinalis* [9]) and allow us to conclude that all *V. officinalis* extracts exhibit a strong ability for the reduction of free radical forms. Considering the reducing power of the samples tested, the activity increased with raising concentrations, and the calculated ASE/mL values obtained were the highest at the concentration of 2 mg/mL. Moreover, similarly to our previous tests, at the concentrations of 1 and 2 mg/mL, the reducing power of the samples was slightly higher than that of BHT [19].

The results of the Fe^2+^ chelating activity assay showed that the most effective extract was characterized by the calculated IC_50_ value = 0.767 mg/mL [19], while the range of samples tested in our study was between 0.034 and 1.674 mg/mL. The standard chelating agent—EDTA (IC_50_ value was 0.007 mg/mL), which was in accordance with the literature data (0.0067–0.017 mg/mL) [19,60].

The results of the antioxidant activity shown by *V. officinalis* extracts are of great importance not only for the prevention of the destructive activity of reactive oxygen species but also for the ability to bind toxic substances, including metal ions, which are nowadays one of the most significant problems affecting human health. As shown above, the confirmed activity of the samples is extending and will surely ensure the efficiency of the extracts as multifunctional bioactive compounds.

#### 3.3.2. Antibacterial Activity

The results of the antimicrobial activity of *V. officinalis* extracts, expressed as MIC and MBC against selected pathogenic bacteria, are shown in Table 5. The MIC values of the tested extracts ranged from 0.3 to 4.5 mg/mL and the MBC values from 0.6 to 18.0 mg/mL. The extracts showed stronger bacteriostatic and bactericidal activity against Gram-positive bacteria (MIC 0.3–2.2 mg/mL, MBC 0.6–9.0 mg/mL) and weaker activity against Gram-negative bacteria (MIC 0.6–9.0 mg/mL, MBC 0.6–18.0 mg/mL). The highest percentage of antibacterial activity (A%) was found for the Vo-LS extract, which inhibited the growth of all tested bacterial strains at a concentration of 2.2 mg/mL (Table 6). Vo-A, Vo-LA, and Vo-RITA extracts inhibited 100% of the tested strains at a concentration of 4.5 mg/mL, while Vo-in vivo extract exhibited an inhibitory effect only at 18.0 mg/mL.

The tested strains differed in their sensitivity to *V. officinalis* extracts. Gram-positive *S. epidermidis* was the most sensitive to all extracts (MIC 0.3–1.1, MBC 0.6–9.0 mg/mL). In turn, Gram-negative *E. aerogenes* and *E. coli* were the most resistant (MIC 1.1–4.5, MBC 2.2–18.0 mg/mL).

The antimicrobial activity of the extracts was also found to differ. The Vo-LS extract showed the best antimicrobial activity of all the tested *V. officinalis* extracts due to the lowest and equal MIC values for both Gram-positive and Gram-negative strains (MIC 0.6–1.1 mg/mL), except in the case of *E. coli*.

The Vo-A, Vo-LA, and Vo-RITA extracts showed the same degree of antimicrobial activity against the majority of Gram-negative strains (MIC/MBC from 1.1 to 4.5 mg/mL), but differences in activity against Gram-positive strains, except for *L. monocytogenes*. Of these three extracts, Vo-RITA was characterized by stronger activity against Gram-positive strains.

Generally, no differences between bacteriostatic and bactericidal activity (MIC and MBC values) were observed for the extracts. Only for *S. epidermidis* and *S. aureus* strains treated with Vo-A, Vo-LS, and Vo-RITA extracts, the MBC values were found to be twice as high as the MIC values. The Vo-in vivo extract showed the weakest bactericidal activity (MBC 4.5–18.0) and the greatest differences between the MIC and MBC values. These results correlate with the lowest content of verbascoside and isoverbascoside in this extract (Table 5 and Table 6).

A comparison of the MIC/MBC values showed that the antibacterial activity of extracts decreased in the following order: Vo-LS > Vo-A = Vo-LA = Vo-RITA > Vo-in vivo.

Compared to the previously tested callus cultures [20], the extracts from microshoot cultures showed higher bacteriostatic and bactericidal activity by approx. 60% (MIC) and 80% (MBC). The dominant biologically active compounds found in the extracts of *V. officinalis* grown in in vitro conditions were verbascoside and isoverbascoside. Lima et al. [61] showed high effectiveness (MIC = 0.6 mg/mL) of the mixtures of verbascoside and isoverbascoside against various bacteria such as *S. aureus*, *P. aeruginosa* *Bacillus subtilis*, *Enterococcus faecalis*, and *E. coli* [62], which indicated higher effectiveness of verbascoside than isoverbascoside against Gram-positive bacteria (*S. aureus* and *E. faecalis*) compared to Gram-negative bacteria (*E. coli* and *P. aeruginosa*). Funes et al. [63] reported that the biological activity of verbascoside may be related to its ability to modulate membrane-dependent cellular processes. Avila et al. [64] provided a detailed description of the action of verbascoside on bacterial cells and indicated that verbascoside inhibits protein production by impeding leucine absorption.

## 4. Conclusions

The presented study showed that the main group of metabolites accumulated in the established different types of *V. officinalis* microshoot cultures were phenylpropanoid glycosides with verbascoside as a quantitative dominant compound (more than 4.7 g/100 g DW). Among them, Vo-LA cultures showed the highest concentration of verbascoside (4.88 g/100 g DW) and also a very high Gi (1027%), which proves that this type of culture can be a potential source of verbascoside.

The studies proved that the high content of phenylpropanoid glycosides and phenolic acids, as well as the total phenolic content, are associated with strong antioxidant and antibacterial activities.

The results of the DPPH assay showed that Vo-A extract was found to be the most effective. The highest radical scavenging activity was found in the extract of Vo-LA cultures, and the most effective reducing power was observed in Vo-A cultures. The extract from the biomass of Vo-Rita bioreactors showed the highest chelating activity. The results of antioxidant assays proved that extracts from established and tested by us microshoot culture extracts were comparable or stronger than those studied for comparison with soil-grown plant extracts.

The tested in vitro culture extracts showed significantly stronger bacteriostatic and bactericidal activity against Gram-positive bacteria and weaker activity against Gram-negative bacteria. The extract from the Vo-LS microshoot culture showed the strongest antibacterial activity, while the extract from the soil-grown plant showed the least activity.

The results of the present study provide fundamental information about the chemical composition and biological activities of *V. officinalis* microshoot cultures maintained in various in vitro systems. We compared the material obtained by biotechnological methods to the herb collected from in vivo conditions. The results indicate that in vitro culture can be a potential alternative to the plant’s raw material.

Additionally, knowledge of the influence of different in vitro systems on the chemical profiles of plant cultures was proved, under our study, can be useful for the effective production of secondary metabolites by biotechnological methods. Our findings may provide a valuable basis for further development of in vitro plant cultures as a source of bioactive compounds for, e.g., pharmaceutical and cosmetic industries.

## Figures and Tables

**Table 1:** Phenylpropanoid glycosides identified by UHPLC-HR-QTOF-MS.

R_t_ (min)	Analyte
4.3	Verbascoside M+ Na+: 647.1932, 471.1488, 325.0914, 163.0381
4.5	Isoverbascoside M + Na+: 647.1932, 471.1488, 325.0914, 163.0381
4.7, 4.9, 5.1	Leucoseptoside A/isomers M+ Na+: 661.2083, 485.1635, 339.1034, 177.0533
5.6, 5.8	Cistanoside D/isomer M+ Na+: 675.2227, 485.1636, 339.1056, 177.0532

**Table 2:** Verbascoside and isoverbascoside concentrations (mg/100 g DW ± SD) measured in extracts from *V. officinalis* in vitro biomass and from plant material.

Extract	Verbascoside	Isoverbascoside
Vo-A	4818.22 ± 79.89	330.56 ± 0.17
Vo-LS	4777.11 ± 5.23	264.90 ± 1.33
Vo-LA	4881.61 ± 99.7	451.80 ± 2.85
Vo-RITA	4722.20 ± 142.74	266.50 ± 2.32
Vo-in vivo	1728.97 ± 29.78	78.34 ± 3.28

**Table 3:** Phenolic acid concentrations (mg/100 g DW ± SD) and total phenolic content measured in extracts of *V. officinalis* in vitro cultures; agar (Vo-A), stationary liquid (Vo-LS), agitated (Vo-LA) and bioreactor cultures (Vo-RITA) and from plant material (Vo-in vivo).

Extract	Ferulic Acid	Protocatechuic Acid	Rosmarinic Acid	Total Phenolics [mg GAE/g DW]
Vo-A	8.35 ± 0.54	3.76 ± 0.07	nd	163.58 ± 1.28
Vo-LS	17.19 ± 0.48	5.30 ± 0.19	nd	137.96 ± 4.25
Vo-LA	23.69 ± 0.21	7.59 ± 0.48	nd	122.93 ± 2.08
Vo-RITA	16.66 ± 0.75	3.51 ± 0.08	nd	129.25 ± 1.63
Vo-in vivo	29.76 ± 2.51	25.75 ± 1.65	2.53 ± 0.11	137.51 ± 3.60

nd—not detected.

**Table 4:** Determination of free radical scavenging activity (DPPH test), reducing power, and ferrous ion (Fe^2+^) chelating activity of extracts of *V. officinalis* in vitro cultures; agar (Vo-A), stationary liquid (Vo-LS), agitated (Vo-LA) and bioreactor cultures (Vo-RITA) and of plant material (Vo-in vivo).Values are expressed as mean ± SD (n = 3).

Extract	DPPH Test, IC_50_ [mg/mL]	Reducing Power Assay [ASE/mL]	Fe^2+^ Chelating Activity, IC_50_ [mg/mL]
Vo-A	0.081 ± 0.036	4.215 ± 0.006	0.110 ± 0.037
Vo-LS	0.184 ± 0.051	3.048 ± 0.006	0.085 ± 0.020
Vo-LA	0.313 ± 0.022	2.452 ± 0.003	0.182 ± 0.036
Vo-RITA	0.125 ± 0.060	2.684 ± 0.005	0.034 ± 0.017
Vo-in vivo	0.214 ± 0.011	2.800 ± 0.005	1.674 ± 0.023
Standard	BHT 0.060 ± 0.003	BHT 2.776 ± 0.005	EDTA 0.007 ± 0.002

**Table 5:** MIC (MBC) values (mg/mL) of extracts of *V. officinalis* in vitro cultures; agar (Vo-A), stationary liquid (Vo-LS), agitated (Vo-LA) and bioreactor cultures (Vo-RITA) and of plant material (Vo-in vivo).

*V. officinalis* Extract	Gram-Positive Bacteria				Gram-Negative Bacteria							
	*S. epidermidis*	*S. aureus*	*B. cereus*	*L. monocytogenes*	*Y. enterocolitica*	*P. aeruginosa*	*K. pneumoniae*	*P. mirabilis*	*Sh. sonnei*	*S. enteritidis*	*E. aerogenes*	*E. coli*
Vo-A	0.3 (0.6)	1.1 (2.2)	2.2 (2.2)	2.2 (2.2)	1.1 (1.1)	1.1 (1.1)	1.1 (1.1)	1.1 (1.1)	2.2 (2.2)	2.2 (2.2)	4.5 (4.5)	4.5 (4.5)
Vo-LS	0.6 (1.1)	1.1 (1.1)	1.1 (2.2)	1.1 (2.2)	0.6 (0.6)	0.6 (0.6)	1.1 (1.1)	1.1 (1.1)	1.2 (2.2)	1.1 (1.1)	1.1 (2.2)	2.2 (2.2)
Vo-LA	1.1 (1.1)	1.1 (1.1)	2.2 (2.2)	2.2 (2.2)	1.1 (1.1)	1.1 (1.1)	1.1(1.1)	2.2 (2.2)	2.2 (2.2)	2.2 (2.1)	4.5 (4.5)	4.5 (4.5)
Vo-RITA	0.6 (1.1)	0.6 (1.1)	1.1 (1.1)	2.2 (2.2)	2.2 (2.2)	1.1 (1.1)	2.2 (2.2)	2.2 (2.2)	2.2 (2.2)	2.2 (2.2)	4.5 (4.5)	4.5 (4.5)
Vo-in vivo	0.6 (9.0)	1.1 (9.0)	2.2 (4.5)	2.2 (9.0)	1.1 (4.5)	1.1 (9.0)	1.1 (4.5)	2.2 (9.0)	2.2 (4.5)	4.5 (9.0)	4.5 (9.0)	4.5 (18)

**Table 6:** Percentage of antibacterial activity (A%) of extracts of *V. officinalis* in vitro cultures; agar (Vo-A), stationary liquid (Vo-LS), agitated (Vo-LA) and bioreactor cultures (Vo-RITA) and of plant material (Vo-in vivo).

MIC [mg/mL]	Vo-A	Vo-LS	Vo-LA	Vo-RITA	Vo-In Vivo
0.3	8	0	0	0	0
0.6	8	25	0	17	8
1.1	50	92	42	33	42
2.2	83	100	83	83	75
4.5	100	100	100	100	100

## Data Availability

Data is contained within the article.
